# Elevated levels of monocyte-lymphocyte ratio and platelet-lymphocyte ratio in adolescents with non-suicidal self-injury

**DOI:** 10.1186/s12888-022-04260-z

**Published:** 2022-09-19

**Authors:** Qi Zheng, Jin Liu, YaJuan Ji, Yan Zhang, XinChao Chen, BangShan Liu

**Affiliations:** 1 Department of Psychology, Xiamen Xianyue Hospital, Xiamen, 361000 Fujian China; 2 Department of Psychiatry, and National Clinical Research Center for Mental Disorders, The Second Xiangya Hospital of Central South University, Changsha, 410011 Hunan China

**Keywords:** Adolescents, Non-suicidal self-injury (NSSI), Neutrophil–lymphocyte ratio (NLR), Monocyte-lymphocyte ratio (MLR), Platelet-lymphocyte ratio (PLR), Inflammation

## Abstract

**Background:**

Neutrophil–lymphocyte ratio (NLR), monocyte-lymphocyte ratio (MLR), and platelet-lymphocyte ratio (PLR) are blood indicators of systemic inflammation. This study aims to compare the levels of inflammatory indicators derived from blood routine tests between adolescents with non-suicidal self-injury (NSSI) and those with non-NSSI.

**Methods:**

A total of 201 adolescents with mood or emotional disorders were enrolled in this study, among which 106 had engaged in NSSI and 95 had never engaged in NSSI. NLR, MLR, and PLR were calculated based on the complete blood cell count.

**Results:**

There was no significant difference in demographic data between the two groups. The NSSI group exhibited significantly higher MLR (*P* = 0.001) and PLR (*P* = 0.007) than the non-NSSI group. Multivariate logistic regression analysis revealed that MLR (OR 1.545, 95%CI [1.087–2.281], *P* = 0.021) and PLR (OR 1.327, 95%CI [1.215–1.450], *P* < 0.001) were independently associated with NSSI. Receiver operating characteristic (ROC) curve analyses demonstrated that for differentiating NSSI from non-NSSI, the optimal cut-off value of MLR was 0.135 and the area under curve was 0.638 ([0.561- 0.715], *P* < 0.001), with a sensitivity of 90.60% and a specificity of 33.70%; the optimal cut-off value of PLR was 127.505 and the area under curve was of 0.611 ([0.533–0.689], *P* < 0.001), with a sensitivity of 39.60% and a specificity of 81.10%.

**Conclusions:**

Systemic inflammation, as indicated by elevated MLR and PLR, was found to be strongly associated with NSSI among adolescents.

## Background

Non-suicidal self-injury (NSSI) is defined as the direct and deliberate damage to one’s own body for reasons not socially accepted, with no observable intention to die as a consequence of the behavior [1]. It has become a proposed diagnosis in the 5th edition of the Diagnostic and Statistical Manual of Mental Disorders (DSM-5) [2]. A systematic review has shown that the prevalence of NSSI is 7.5 to 46.5% in adolescents [3]. Another meta-analysis found that the lifetime and 12-month prevalence of NSSI was 22.1 and 19.5%, respectively, in children and adolescents between 1989 and 2018 across the world [4]. According to the latest statistics, Chun W et al. estimated that the prevalence of NSSI in middle school students in China (aged 13–18 years) is 27.4% [5]. Numerous studies have shown that NSSI is strongly associated with suicide attempts (SA) [6, 7] and that NSSI is the strongest predictor of future suicidal behavior [8]. The risks of both SA and completed suicide are significantly higher in those who have engaged in NSSI. Among those with any NSSI, 39.6% endorsed suicidal behavior, and 66.3% of those with any suicidal behavior reported NSSI [9]. Therefore, NSSI has been recognized as a major public health problem in adolescents that seriously affects their mental health and safety.

Until very recently, only very few studies investigated the relationship between NSSI and inflammation. Kim et al. reported that patients with mood disorders with NSSI exhibit greater behavioral impulsivity and higher levels of serum tumor necrosis factor α (TNF-α) as compared to those without NSSI [10]. Osimo et al. suggested that low-grade systemic inflammation (defined as a serum C-reactive protein (CRP) level > 3 mg/L or white cell count (WBC) > 9.4^*^10^9^/L) is associated with NSSI in adult psychiatric inpatients through an analysis of electronic health records [11]. However, results are conflicting among different studies. Russell et al. found no association between interleukin-6 (IL-6) and NSSI; they even found a negative association between CRP and NSSI, indicating that higher levels of CRP may be protective for NSSI [12]. Despite the inconsistent findings, there is a growing interest in the relationship between inflammation and NSSI.

According to previous studies, Neutrophil–Lymphocyte Ratio (NLR), Monocyte-Lymphocyte Ratio (MLR), and Platelet-Lymphocyte Ratio (PLR) are relatively stable biomarkers of systemic inflammation [13, 14]. The testing of these markers is cost-effective and easy to access, indicating its potential value for clinical application. Studies have shown that these markers are not only early indicators of severe COVID-19 cases[15], but also closely associated with a variety of diseases, such as cancers [16], cardiovascular disease [17], digestive disease [18] and diabetes mellitus [19, 20]. The markers have also been extensively examined in studies on psychiatric disorders. Previous studies have found low-grade systemic inflammation in adult psychiatric inpatients, especially for those diagnosed with schizophrenia and bipolar disorder (BD) [11]. According to an updated systematic review and meta-analysis of major depressive disorder (MDD) on patient groups in China, the NLR, PLR, and MLR were all elevated in patients with depression, as compared with healthy controls [21]. These markers are also involved in the inflammatory pathophysiology among children and adolescents with psychiatric disorders. According to some studies, the NLR, PLR and MLR of children with attention-deficit hyperactivity disorder (ADHD) or children and adolescents with anxiety disorders (AD) were significantly higher than their healthy counterparts [22, 23]**.** Furthermore, elevated NLR and PLR were found to be associated with inflammation among adolescents with obsessive–compulsive disorder, and those with comorbid anxiety exhibited increased inflammatory responses [24]. A study also found that adolescents with depression had higher NLR, which was positively correlated with the severity of depression [25].

Many studies have investigated the association between NLR, MLR, and PLR and suicide. Ekinci et al. suggested that NLR may be a marker for suicide vulnerability in patients with MDD [26]. one study also showed that NLR in patients with bipolar disorder and SA is higher than that in healthy controls [27]. Similarly, another research also supports that SA survivors exhibited higher levels of NLR compared to normal controls [28]. Moreover, Arafat et al. revealed that the levels of CRP and NLR were significantly elevated in individuals who committed non-fatal hanging [29]. Puangsri et al. found that depressed adolescents with SA show elevated levels of MLR than those with no suicide ideation; and that the levels of MLR and NLR are positively correlated with suicidality severity [30].

To our knowledge, no studies have directly investigated the correlation between blood cell counts and NSSI. Herein we report an investigation on the associations between MLR, NLR, and PLR in adolescents with NSSI. Based on the previous findings, we hypothesized that patients with NSSI would show elevated levels of NLR, MLR, and PLR as compared with those with non-NSSI; and NLR, MLR, and PLR may be effective markers for differentiating patients with NSSI and those with non-NSSI.

## Methods

### Participants

A total of 201 inpatient adolescents (aged 13–18 years) with mood or emotional disorders were recruited from the hospital. The inclusion criteria were: (1) diagnosed with mood or emotional disorders according to the International Classification of Diseases 10^th^ Revision (ICD-10), including anxiety disorders (AD), major depressive disorder (MDD), bipolar disorder (BD), and behavioral and emotional disorders with onset usually occurring in childhood and adolescence; (2) engaged in NSSI according to the DSM-5 criteria (history of NSSI was determined using information collected through the direct interviews and medical record reviews), and having ≥ 1 self-injury at least one occasion in the past month. The participants were grouped into an NSSI group and a non-NSSI group based on whether they had a history of NSSI.

The exclusion criteria were: (1) with a current diagnosis of any other psychiatric disorder; (2) with SA or suicide ideation (assessed based on the patient’s history and course of the disease); (3) with serious physical illnesses, especially those associated with systemic inflammatory responses such as active infection, fever, acute or chronic endocrinological diseases; (4) had used anti-inflammatory drugs, corticosteroids or antipyretic drugs in the past two weeks.

### Data collection

Blood samples of all enrolled individuals were collected from the cubital vein after fasting. Complete blood cell count was measured using Sysmex XN-1000 (XN-1000, Sysmex, Japan) automatic blood cell analyzer. Data were obtained from the electronic medical records system. Laboratory results were obtained from electronic medical records, and NLR, MLR, and PLR values were calculated and compared between the two groups.

### Statistical analysis

Data analyses were performed using the IBM SPSS Statistics version 26 for Mac OS. The normality of data was tested using Kolmogorov–Smirnov test. Normally distributed data were presented as means and standard deviations, and were compared between the two groups using independent two-sample t test. Inter-group comparisons of skewed variables were performed using Mann–Whitney U test. Multivariate logistic regression analysis was conducted to determine the variables independently associated with NSSI. Odds ratios (ORs) and 95% confidence interval (CI) were calculated for the independently associated variables. Receiver operating characteristic (ROC) curve analysis was performed to determine the discrimination threshold of inflammatory markers in the diagnosis of NSSI. A two-tailed *P* < 0.05 was considered statistically significant for all analyses.

## Results

A total of 201 patients were enrolled in this study, with 106 grouped into the NSSI group and 95 grouped into the non-NSSI group. In the NSSI group, there were 89 females and 17 males, with a mean age of 15(14, 16) years; among which 4 (3.77%) patients were diagnosed with anxiety disorders (AD), 56 (52.83%) with major depressive disorder (MDD), 3 (2.83%) with bipolar disorder (BD) and 43 (40.57%) with behavioral and emotional disorders with onset usually occurring in childhood and adolescence. In the non-NSSI group, there were 74 females and 21 males, with a mean age of 15(14, 17) years; among which 9 (9.47%) patients were diagnosed with AD, 42 (44.21%) with MDD, 5 (5.26%) with BD and 39 (41.05%) with behavioral and emotional disorders with onset usually occurring in childhood and adolescence. There were no significant differences in age (*p* = *0.094*), gender (*p* = *0.359*), family history (*p* = *0.125*), and diagnoses (*p* = *0.260*) between the two groups. The demographic characteristics of the subjects are summarized in Table 1.

**Table 1:** Demographic characteristics of the NSSI group and the non-NSSI group

Variables	NSSI (*n* = 106)	Non-NSSI (*n* = 95)	z/x^2^	*p*
Age (y)	15(14, 16) ^a^	15(14, 17) ^a^	-1.676	0.094^b^
Female (n%)	89(84.00%)	74(77.90%)	0.839	0.359^c^
Family history	5(4.70%)	11(11.60%)	2.351	0.125
Diagnosis			4.030	0.260
Anxiety disorders	4(3.77%)	9(9.47%)		
Major depressive disorder	56(52.83%)	42(44.21%)	-	-
Bipolar disorder	3(2.83%)	5(5.26%)	-	-
Behavioral and emotional disorders with onset usually occurring in childhood and adolescence	43(40.57%)	39(41.05%)	-	-

^a^ Expressed as median (lower–upper quartiles)

^b^ Mann–Whitney U test

^c^ Chi-square test

The monocyte count, MLR, and PLR were significantly higher in adolescents with NSSI than those without NSSI (*p* = *0.011, p* = *0.001, p* = *0.007,* respectively). The lymphocyte count was significantly lower in the NSSI group (*p* = *0.043*). There were no significant differences in white blood cell count (WBC), neutrophil count, platelet count, mean platelet volume (MPV), and NLR between the two groups (all *p* > *0.05*) (Table 2). Multivariate logistic regression analysis showed that MLR (OR 1.545, 95% CI [1.087–2.281], *p* = 0.021) and PLR (OR 1.327, 95%CI [1.215–1.450], *p* < 0.001) were independently associated with NSSI (Table 3).

**Table 2:** Comparison of blood cell count parameters between the NSSI group and the non-NSSI group

Variables	NSSI	Non-NSSI	t/z	*p*
WBC (10^3^/uL)	6.61 (5.92, 7.64) ^a^	6.68 (5.72, 8.03) ^a^	-0.118	0.906^b^
Lymphocyte (10^3^/uL)	2.39 (2.02, 2.68)	2.56 (2.03, 3.16)	-2.025	0.043^*^
Monocyte (10^3^/uL)	0.46 (0.39, 0.56)	0.42 (0.34, 0.51)	-2.531	0.011^*^
Neutrophil (10^3^/uL)	3.61 (2.80, 4.19)	3.41 (2.46, 4.76)	-0.730	0.465
Platelet (10^3^/uL)	288.64 ± 56.08^d^	273.55 ± 59.08^d^	1.852	0.066^e^
MPV (fl)	9.94 ± 0.75	10.11 ± 0.93	-1.428	0.155
MLR	0.19 (0.16, 0.23)	0.16 (0.13, 0.20)	-3.381	0.001^*^
NLR	1.46 (1.13, 1.94)	1.34 (0.96, 1.87)	-1.690	0.091
PLR	115.66 (97.63, 146.72)	107.85 (88.89, 125.37)	-2.709	0.007^*^

*WBC* White blood cell count, *MPV* Mean platelet volume, *MLR* Monocyte-lymphocyte ratio, *NLR* Neutrophil–lymphocyte ratio, *PLR* Platelet-lymphocyte ratio

^a^ Expressed as median (lower–upper quartiles)

^b^ Mann–Whitney U test

^d^ Expressed as mean ± standard deviation

^e^ Student’s t-test

^*^ indicates statistical significance

**Table 3:** Multivariate logistic regression model for the association between NSSI and inflammatory marker

Variables	OR (95%CI)	*p*
MLR	1.545(1.087–2.281)	0.021^*^
PLR	1.327(1.215–1.450)	< 0.001^*^

*MLR* Monocyte-lymphocyte ratio, *PLR* Platelet-lymphocyte ratio

^*^ indicates statistical significance

**Fig. 1:**
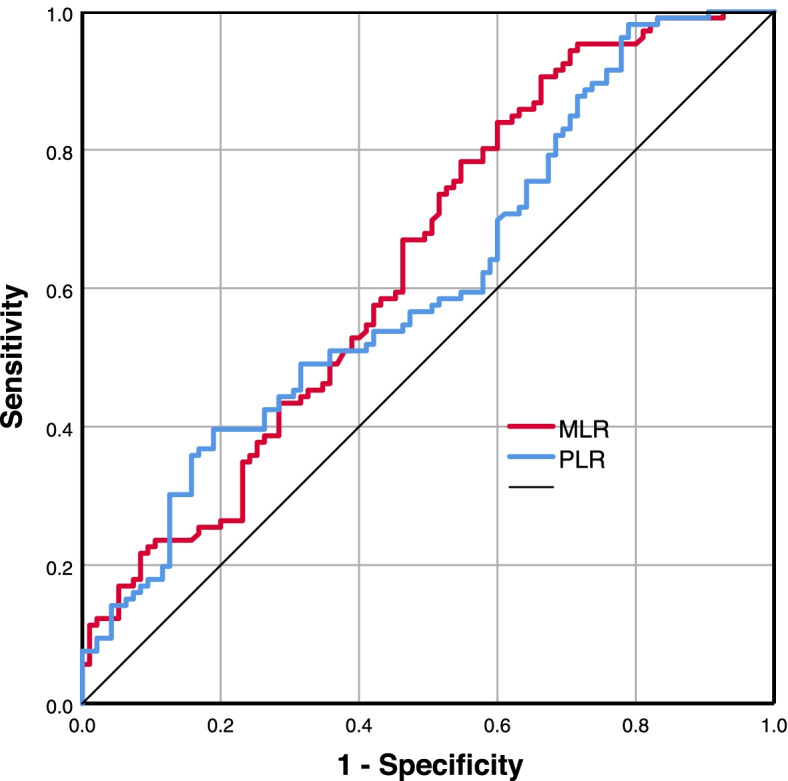
The ROC curves of MLR and PLR for differentiating NSSI from non-NSSI. MLR is presented with a red line, with an AUC of 63.8%, a sensitivity of 90.60%, and a specificity of 33.70%. PLR is presented with a blue line, with an AUC of 61.1%, a sensitivity of 39.60%, and a specificity of 81.10%

Receiver-operating characteristic (ROC) curve analyses were then performed to evaluate the diagnostic ability of MLR and PLR for NSSI. According to the ROC curve, the area under curve was 0.638 ([0.561- 0.715], *p* < *0.001*) for MLR and 0.611 ([0.533–0.689], *p* < *0.001*) for PLR. The optimal cut-off value was 0.135 for MLR, with a sensitivity of 90.60% and specificity of 33.70%; the optimal cut-off value was 127.505 for PLR, with a sensitivity of 39.60% and specificity of 81.10% (Table 4 and Fig. 1**)**.

**Table 4:** Receiver-operating characteristic (ROC) curve of MLR and PLR for differentiating NSSI from non-NSSI

Variables	AUC	95%CI	Cut-off	sensitivity	specificity	*p*
MLR	0.638	0.561–0.715	0.135	0.906	0.337	< 0.001^*^
PLR	0.611	0.533–0.689	127.505	0.396	0.811	< 0.001^*^

*MLR* Monocyte-lymphocyte ratio, *PLR* Platelet-lymphocyte ratio

^*^ indicates statistical significance

## Discussion

To the best of our knowledge, this is the first study probing NLR, MLR, and PLR as hallmarks of systemic inflammation in adolescents with NSSI. The results showed significantly elevated levels of MLR and PLR in adolescents with NSSI compared with those with no NSSI, suggesting a potential role of MLR and PLR in the assessment of NSSI in clinical practice. Inconsistent with our hypothesis, we found no changes in NLR in adolescents with NSSI.

### MLR

Previous studies have found that an elevated level of monocyte count is associated with overexpression of immune genes and overproduction of monocytes/macrophage-related cytokines [31]. Increased monocyte count or MLR had been observed in major psychiatric disorders, such as schizophrenia [13, 32], bipolar disorder [33, 34] and major depressive disorder [35]. It is supposed that elevated monocyte count or MLR is accompanied by an increase in the monocyte-related cytokines (such as IL-6, IL-1β, IL-12, and TNF) and activation of microglia in the brain [36], which is a biomarker of neuroinflammation. Microglia monitors the functional state of synapses and modulates neuroplasticity by synaptic pruning and phagocytosis, resulting in altered brain structure and function, including regions implicated in impulse control and emotion regulation, such as the medial prefrontal cortex, dorsolateral prefrontal cortex, insula, amygdala, and hippocampus [7, 37–39]. A previous study found that elevated monocyte count and MLR are associated with an increased risk of suicide in adolescents with major depressive disorder [30]. In sum, the elevated monocyte and MLR found in this study may play an important role in the pathology of NSSI through the release of monocyte-related cytokines and activation of microglia.

### PLR

Platelets are closely related to stress, peripheral inflammation, and monoaminergic neurotransmission, which may be involved in the pathology of NSSI. Stress may increase platelet count and activity through sympathetic system activation [40]. The increased platelet count and activity may further induce altered endothelial permeability as well as recruitment of neutrophils and macrophages, resulting in elevated peripheral inflammation [41, 42]. Moreover, platelets contain abundant serotonin in their dense granules, and serotonin receptors (5HT_2A_) and transporters on the cell surface, are involved in the production, reuptake, and metabolism of serotonin [43, 44]. A previous study had suggested that platelet serotonin could reliably indicate the presynaptic serotonin activity in the brain [45].

Dysregulation of serotonergic neurotransmission is an important factor linked to emotion and behavior disorders, particularly impulse control disorders [46–48]. As reported in a review paper, NSSI could be considered a behavior associated with impulse control problems [49]. Previous studies found that the aggressivity and impulsivity of suicidal attempters seem to be associated with the serotonin content in platelets and the total number of 5-HT_2A_ receptors on platelet surface [50, 51]. Similarly, E. Gorodetsky et al. found the serotonin transporter gene polymorphism (5-HTTLPR) were strongly associated with in prisoners with NSSI [52]. Similar to human behavioral data, preclinical studies also revealed a close relationship between serotonergic neurotransmission and NSSI. Dellu et al. revealed that impulsivity behavior was mainly associated with decreased serotonergic neurotransmission in the amygdala and anterior cingulate cortex by testing the different behavior of rats [53]. A recent study in the nonhuman primate model showed that primates with the s allele exhibited NSSI and impulsive behaviors, and there may be distinct the serotonin transporter (5-HTT) genotype-mediated different NSSI typologies [54]. Taken together, elevated PLR may play a role in the pathophysiology of NSSI through its influence on peripheral inflammation and serotonin neurotransmission.

### NLR

Inconsistent with our hypothesis, we found no significant difference in NLR between adolescents with NSSI and those with no NSSI, despite a decrease in the lymphocyte count. NLR reflects the balance between innate (neutrophils) and adaptive (lymphocytes) immunity and is considered a stable peripheral biomarker of inflammation [55]. Previous studies have found that NLR is correlated with suicidality in major depression [26, 56]. The reason for the nonsignificant finding of NLR in this study remains unclear. Puangsri et al. suggested that MLR is more closely associated with SA in patients with MDD than NLR and PLR [30]. Turkmen et al. found that PLR is superior to NLR in denoting inflammation in patients with end-stage renal diseases [57]. Thus, our result may indicate that MLR and PLR are superior to NLR in the assessment of NSSI in adolescents.

### Cut-off values of MLR and PLR

This study for the first time suggested cut-off values of MLR and PLR for differentiating NSSI from non-NSSI. With a cut-off value of 0.135, the ROC analysis of MLR reached a high sensitivity (90.60%) and low specificity (33.70%); with a cut-off value of 127.505, the ROC analysis of PLR reached a high specificity (81.10%) and low sensitivity (39.60%). Despite the relatively low areas under the curve for MLR (0.638) and PLR (0.611), a combination of MLR and PLR would be promising to improve the overall sensitivity and specificity in differentiating NSSI from non-NSSI.

To be mentioned, a previous study showed that the MLR and PLR values vary with age and gender in the Chinese population, with males exhibiting higher PLR and lower MLR than females, and with age increases, PLR decreases and MLR increases [58]. In our study, females accounted for the majority of the sample, and all of the participants were aged ≤ 18. Thus, the findings of the ROC analyses should be interpreted with caution, as the optimal cut-off values obtained from our sample maybe not be suitable for males or other age groups. Future studies should investigate the cut-off values of MLR and PLR for differentiating NSSI from non-NSSI with stratification of gender and age.

The present study suggested that elevated PLR and MLR were associated with NSSI, while some other studies reported that NLR might be a potential new peripheral biomarker of suicidal behavior [26, 56], indicating that the pathological mechanism of the two conditions might be different. Unfortunately, the available therapeutic interventions for NSSI mainly include psychotherapy and adjuvant psychopharmacological treatment [59–61], the effects of which are suboptimal. An updated study suggested that nonsteroidal anti-inflammatory drugs (NSAIDs), such as ibuprofen, naproxen, celecoxib, and aspirin, as compared to acetaminophen, could significantly reduce suicidal ideation [62]. Based on the above findings, it is reasonable to speculate that anti-inflammatory drugs might be helpful in the treatment of NSSI.

### Limitations

There are still some limitations to this study. First, we were not able to control some factors affecting the inflammatory status of participants, such as smoking, alcohol consumption, substance use, menstrual cycle, and body mass index (BMI). Second, the possible association between the severity of NSSI symptoms and inflammatory indicators was not evaluated. Third, other indicators of immune functions, such as CRP and interleukins, were not evaluated. Fourth, the participants were recruited by convenience sampling from one hospital, which may bring about selection bias and decrease the representativeness of the sample. Finally, the present study is a cross-sectional study with a small sample size, which precluded us from identifying any causal relationship between the inflammatory indicators and NSSI.

## Conclusion

Through this study, we have found that systemic inflammation, as indicated by elevated MLR and PLR, was strongly associated with NSSI among adolescents. It is possible that the levels of biomarkers change with NSSI-related processes.

## Data Availability

The datasets generated and/or analyzed during the current study are not publicly available data and anonymity but are available from the corresponding author on reasonable request.

## Abbreviations

NLR: Neutrophil–lymphocyte ratio
MLR: Monocyte-lymphocyte ratio
PLR: Platelet-lymphocyte ratio
NSSI: Non-suicidal self-injury
ROC: Receiver operating characteristic
DSM-5: The Diagnostic and Statistical Manual of Mental Disorders
SA: Suicide attempts
TNF-α: Tumor necrosis factor α
CRP: C-reactive protein
WBC: White cell count
IL-6: Interleukin-6
MDD: Major depressive disorder
BD: Bipolar disorder
ADHD: Attention-deficit hyperactivity disorder
AD: Anxiety disorders
ICD-10: The International Classification of Diseases 10^th^ Revision
ORs: Odds ratios
CI: Confidence interval
MPV: Mean platelet volume
5-HT_2A_: Serotonin receptors
5-HTTLPR: Serotonin transporter gene polymorphism
5-HTT: Serotonin transporter
NSAIDs: Nonsteroidal anti-inflammatory drugs
BMI: Body mass index
